# Designer DNA Architectures: Applications in Nanomedicine

**DOI:** 10.5772/63228

**Published:** 2016-01-01

**Authors:** Arun Richard Chandrasekaran

**Affiliations:** 1 The RNA Institute, University at Albany, State University of New York, USA

**Keywords:** DNA Nanostructures, DNA Origami, Nanomedicine, DNA Nanodevices

## Abstract

DNA has been used as a material for the construction of nanoscale objects. These nanostructures are programmable and allow the conjugation of biomolecular guests to improve their functionality. DNA nanostructures display a wide variety of characteristics, such as cellular permeability, biocompatibility and stability, and responsiveness to external stimuli, making them excellent candidates for applications in nanomedicine.

## 1. Introduction

**Figure 1.**
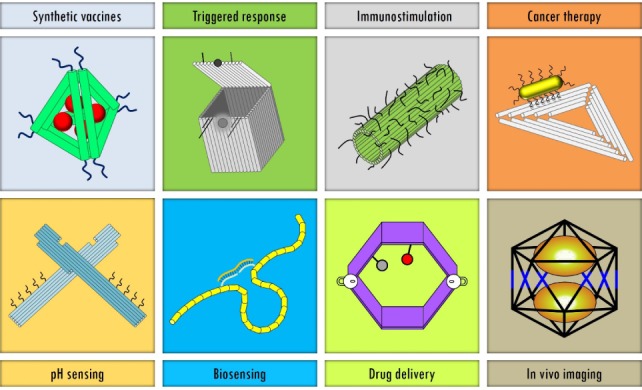
Gallery of DNA nanostructures used for biomedical applications. Illustrations of synthetic vaccine complexes [24], a DNA origami box that responds to external stimuli [31], DNA nanotube/CpG sequences for immunostimulation [23], DNA origami-gold nanorod complexes for cancer therapy [29], DNA origami nanopliers for pH sensing [38], DNA nanoswitches for nucleic acid detection [20], a DNA nanorobot for molecular payloads [32] and a DNA icosahedron for *in vivo* imaging [36].

The impact of nanotechnology in the field of medicine has been profound in the past two decades, with new nanoscale materials, such as nanofibres [1], liposomes [2] and nanoparticles [3], having been developed for use in a variety of biomedical applications. DNA is one such material that has found an exemplary use within nanomedicine. Apart from its well-known role as a genetic material, DNA has been shown to be valuable as a molecular building block for the construction of nanoscale objects [4]. With its diameter of ∼2 nm and a helical pitch of ∼3.4–3.6 nm, DNA is inherently a nanoscale material. Moreover, the highly specific Watson-Crick base pairing (A:T and G:C) provides a molecular recognition system for designing complex structures using DNA [5]. In addition, sticky end cohesion [6] provides control over the programmable assembly of hierarchical structures based on DNA motifs [7]. While DNA motifs can be used to construct smaller objects, the DNA origami technique [8] has been used to create custom-made DNA nanostructures that are hundreds of nanometres in size. These features of DNA have made it useful in the construction of a variety of materials, such as two- [9] and three-dimensional lattices [10–13], topologically linked arrays [14–15], nanomachines [16–18] and biosensors [19–20]. These novel DNA materials have been used in a variety of biomedical applications [21], which include the triggered release of cargo, biosensing synthetic vaccines, drug delivery and *in vivo* imaging (Figure 1).

## 2. Applications in Immunostimulation and Drug Delivery

**Figure 2.**
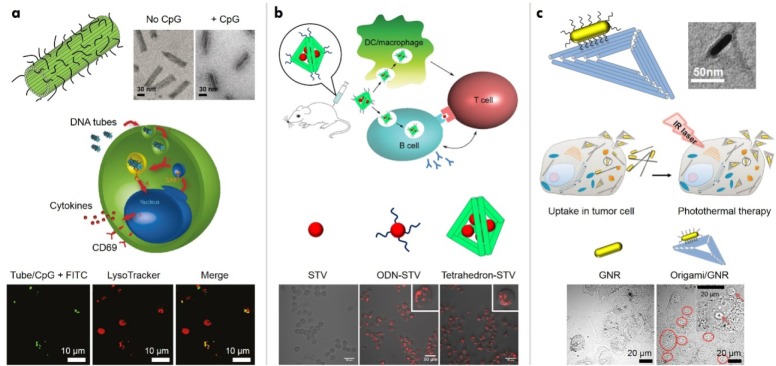
Drug delivery and cancer therapy using DNA nanostructures. (a) DNA origami nanotubes coated with CpG oligonucleotides for immunostimulation. Adapted with permission from [23]. Copyright 2011, American Chemical Society (ACS). (b) DNA tetrahedra with streptavidin/CpG oligonucleotides forming a synthetic vaccine complex Adapted with permission from [24]. Copyright 2012, ACS. (c) DNA origami/gold nanorod (GNR) complex for cancer treatment using photothermal ablation Adapted with permission from [29]. Copyright 2015, Wiley-VCH.

DNA nanostructures exhibit several characteristics that make them promising candidates for applications in nanomedicine. One such aspect is enhanced cellular permeability [22], compared to single-stranded and double-stranded DNA. This feature allows DNA architectures, modified with biologically active molecules, to trigger cellular mechanisms. For example, DNA origami nanostructures containing CpG sequences on their surface were shown to induce a high level of immune response in mammalian cells (Figure 2a) [23]. The DNA nanostructure used in this case was a 30-helix nanotube constructed from an 8634-nucleotide single-stranded DNA scaffold and over 200 staple strands. Some of the staple strands were modified in order to provide extensions that can attach up to 62 CpG sequences. Endosomal uptake of these structures was shown by fluorescence microscopy, and CpG-loaded DNA nanotubes exhibited significantly higher cell permeability. Moreover, these constructs elicited a higher immune response in mouse splenocytes by triggering an immunostimulatory cascade, which was mediated by Toll-like receptor 9 (TLR9). The stability and internalization of these nanotubes show that DNA-based nanostructures are promising delivery vehicles for immune system activation. Such features can also be combined with the functionalization of proteins in order to create antigen-adjuvant complexes. Streptavidin molecules have been functionalized onto tetrahedral DNA nanostructures, along with CpG oligo-deoxynucleotides, for the creation of a synthetic vaccine complex (Figure 2b) [24]. The fully assembled vaccine complex exhibited a higher level of immunogenicity when compared to either an unassembled mixture of streptavidin and CpG sequences or streptavidin alone. Such DNA scaffolds are useful in constructing antigen-adjuvant complexes that elicit a strong and specific antibody response *in vivo*, without inducing an undesirable response against the carrier itself. Other examples of DNA architectures that have been used for immunostimulation include a DNA tetrahedron with CpG motifs [25] and polypod-like structures [26].

The stability of DNA nanostructures in cell lysates from cancerous cell lines [27] has prompted research on the use of these structures for cancer diagnosis and treatment. In one such example, DNA origami structures in the shapes of a triangle, square and tube, were used as carriers of the anticancer drug doxorubicin [28]. The accumulation capacity of different DNA origami shapes in tumour cells was evaluated by tagging the structures with quantum dots and using fluorescence imaging, in which the accumulation level of triangle nanostructures was found to be optimal. Doxorubicin was loaded onto the DNA origami structures by incubating them for 24 hours, with about 50% of the drug estimated to have been loaded onto the nanostructures. These doxorubicin-loaded DNA origami structures exhibited efficient cellular uptake, enhanced tumour selectivity and long-lasting therapeutic effects. Specifically, doxorubicin-loaded origami structures exhibited reduced tumour burden in nude mice, compared to doxorubicin by itself. In addition, gold nanorods functionalized on DNA origami triangles and nanotubes have been used for photothermal cancer therapy *in vitro* and *in vivo* (Figure 2c) [29]. Doxorubicin-loaded DNA origami structures have also been shown to cause high cytotoxicity in human breast adenocarcinoma cancer cells (MCF 7) [30].

**Figure 3.**
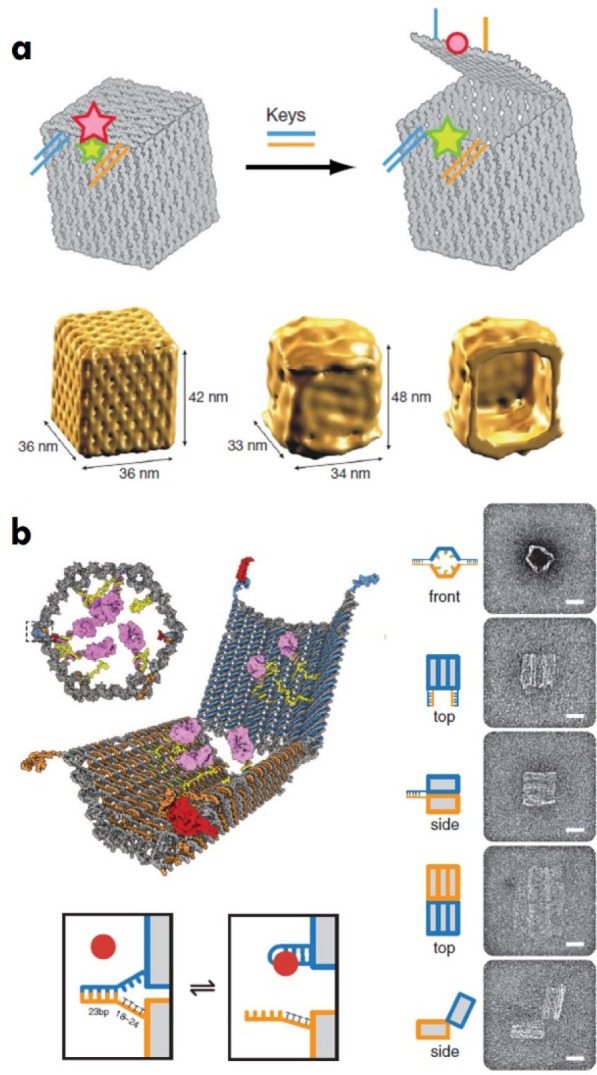
Stimuli responsive DNA nanostructures. (a) A DNA origami box with a controllable lid that can be opened using additional DNA strands. Reproduced with permission from [31]. Copyright 2009, Nature Publishing Group (NPG). Single-particle reconstruction of the DNA box using cryo-EM images is shown at the bottom. (b) A DNA nanorobot that can be triggered open by antigen keys. Reproduced with permission from [32]. Copyright 2012, The American Association for the Advancement of Science. The nanorobot has been used to deliver gold nanoparticles and antibody fragments. TEM images of robots in closed and open conformations are shown on the right. Scale bars: 20 nm.

Another feature of DNA-based nanostructures is that they allow the encapsulation and release of drugs in a controlled fashion, thus opening up new avenues in targeted drug delivery. For example, dynamic DNA nanostructures, such as the DNA origami box [31], can be locked or opened using additional DNA strands and provide a route to the triggered delivery of cargo (Figure 3a). In this design, DNA origami was used to create a six-connected planar structure, which can then fold into the six faces of a cube-like box. The segment on the top, which acts as a lid, was “locked” by the formation of a duplex between the two adjacent segments. One of the strands of this locking duplex contained a single-stranded extension that can act as a toehold. On the addition of a fully complementary “key” DNA strand, the lid is opened via strand displacement of the lock strand. Moreover, DNA origami nanostructures have been used to host cargo molecules for delivery to cells. One such example is a DNA origami nanorobot containing two aptamers, which are specific to two different receptors on the cell membrane (Figure 3b) [32]. This structure is closed by hybridization of the aptamer to a complementary strand, but is opened in the presence of an antigen key (which can bind to the aptamer). This nanorobot was programmed to deliver gold nanoparticles or antibody fragments in response to specific cell receptors. Other examples of DNA nanostructures with the ability to respond to external stimuli are a pH-dependent DNA tetrahedron [33] and a temperature-triggered octahedral DNA cage that can release enzymes [34].

## 3. Applications in Imaging and Biosensing

**Figure 4.**
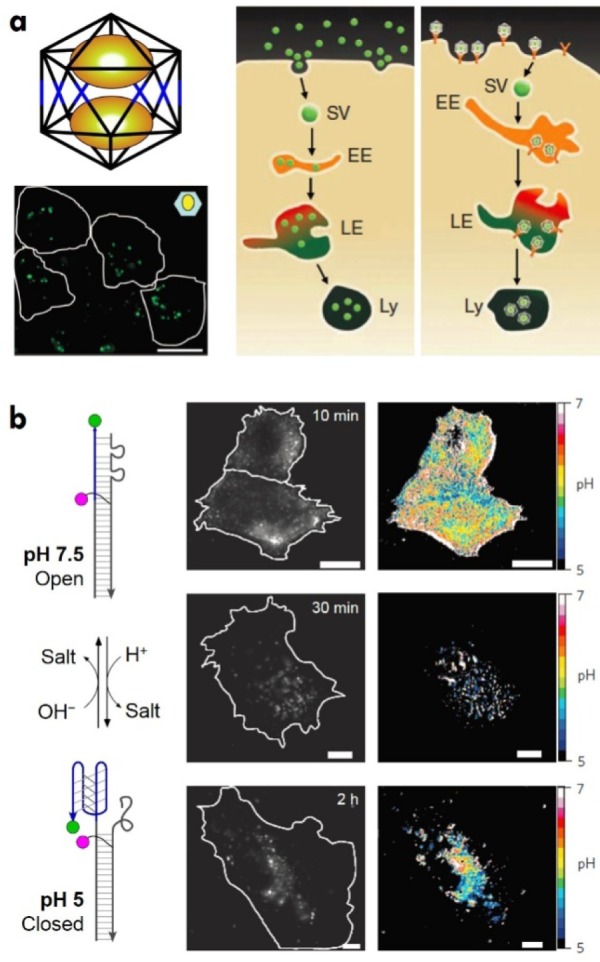
DNA nanostructures for *in vivo* imaging and sensing applications. (a) A DNA icosahedron containing fluorescein isothiocyanate-dextran (FD) as a cargo used for *in vivo* imaging. Adapted with permission from [36]. Copyright 2011, NPG. Also displayed is an image showing internalization of an icosahedron/FD complex in *Drosophila* haemocytes. *Right:* Endocytosis pathways adopted by free and encapsulated FD (EE, early endosome; LE, late endosome; Ly, lysosome; SV, spherical vesicle). (b) A nanomachine based on a DNA i-motif for tracking pH changes in the endosomal furin pathway. Adapted with permission from [17]. Copyright 2013, NPG. Widefield images and corresponding donor-to-acceptor maps at different pulse times are shown on the right.

DNA nanostructures facilitate the attachment of a multitude of components, including fluorophores, making them useful for imaging purposes. Tubular DNA origami with a label-free fluorescent probe (carbazole-based biscyanine molecule) has been used for the direct visualization of their distribution and stability in live, cellular systems [35]. This strategy allowed the monitoring of DNase I digestion of origami nanostructures and the tracking of their localization in lysosomes. DNA polyhedra, encapsulating a fluorescent biopolymer fluorescein isothiocyanate-dextran (FD), have also been used for *in vivo* imaging (Figure 4a) [36]. In this case, five-arm DNA branched junctions were used to create the top and bottom halves of the DNA icosahedron. FD was loaded into the DNA icosahedra and tested in coelomocytes of *Caenorhabditis elegans*. These cells express anionic ligand-binding receptors (ALBR), which internalize negatively charged entities by receptor-mediated endocytosis [37], thereby engulfing higher quantities of FD encapsulated in the DNA icosahedron than FD by itself. Furthermore, the functionality of this host-cargo complex was demonstrated *in vivo* by quantitatively mapping pH changes associated with endosomal maturation. Such programmable host-cargo complexes can be used as functional probes to interrogate biological phenomena, both in living cells and whole organisms.

One other biological application of DNA nanostructures is in chemical and biological sensing [19]. DNA-based biosensors have proven to be cost-effective, sensitive and have the potential to be used as point-of-care diagnostic tools. For example, DNA-based nanoswitches have been used for nucleic acid detection using an easy gel readout [20]. The nanoswitch was designed from an M13 single strand and complementary ‘backbone’ oligonucleotides. Two of these strands can be modified to contain single-stranded extensions, which are partly complementary to the target sequence to be detected. The ‘off’ state of the nanoswitch is a linear duplex, while target binding brings the two detectors closer to form a looped ‘on’ state. The two states of the nanoswitch migrate differently on a gel, thus giving a binary read out. Another example is a DNA origami-based nanoplier [38] used for pH sensing. The two levers of the DNA origami nanoplier were designed to contain a series of C-rich single-stranded extensions. The device works on the basis of i-motif formation between C-rich sequences at acidic pH. For example, the levers remain in the “open” configuration at neutral pH; meanwhile, at acidic pH, the C-rich strands form an intermolecular i-motif, thereby bringing the two levers together into a “closed” configuration. The two structural states can be visualized using an AFM. Intracellular pH sensors have also been developed based on the i-motif (Figure 4b) [17]. This nanomachine, called the “I-switch”, consisted of a long strand forming a duplex with two short strands. The two ends of the long strand contained C-rich single-stranded extensions containing a fluorescent dye. At neutral pH, the three strands remain in the open linear structure (duplex) and exhibit a low FRET signal due to the separation of the two dyes. However, at acidic pH, the single-stranded extensions fold to form an i-motif, thereby bringing the two dyes into close proximity and resulting in a high FRET signal. This system has been used to track real-time pH changes in early endosomes and the trans-Golgi network.

## 4. Conclusion

In summary, DNA nanostructures portray a wide variety of characteristics, such as cellular permeability, biocompatibility, site-specific functionalization of molecules and the ability to respond to external stimuli, which in turn make them excellent candidates for use in biomedical applications [39–40]. A wide variety of structures in sizes ranging from a few nanometres (based on DNA motifs) to hundreds of nanometres and into the micrometre range (based on DNA origami) can be created. Moreover, techniques such as molecular canvas [41] and DNA bricks [42] provide alternate routes to creating nanostructures. In addition, micrometre scale arrays can be created by combining DNA origami and lithography, which allows precise and programmable surface interactions. Such assemblies provide a large surface area for multiplexed diagnostic purposes. Despite advantages, such as spatial positioning of other biomolecules [43] through various conjugation strategies [44], the use of DNA nanostructures also has some limitations. For example, the DNA origami method involves hundreds of staple strands, which is a limiting factor; however, the cost of synthetic DNA used for these purposes has reduced in recent times [45], making DNA a viable material to use for biomedical applications. One other issue is the dependence of the DNA origami technique on single-stranded scaffolds (the viral genome M13 is frequently used), but the creation of custom-made DNA origami scaffolds [46] allows the creation of structures of various sizes in order fit their purpose. Applications in nanomedicine will be aided by further research in the encapsulation of a variety of drugs and biomolecules, conjugation strategies and triggered responses based on external and environmental stimuli. More intensive research is needed for analysing the biocompatibility, cellular uptake and intracellular behaviour of DNA constructs, but they have so far exhibited unparalleled advantages over current strategies for use in nanomedicine.

## 5. Conflict of interest

The author declares no conflict of interest.
